# Elevated Circulating TNF-α in Fat-Free Mass Non-Responders Compared to Responders Following Exercise Training in Older Women

**DOI:** 10.3390/biology3030551

**Published:** 2014-09-05

**Authors:** Gordon Fisher, C. Scott Bickel, Gary R. Hunter

**Affiliations:** 1 Department of Human Studies, University of Alabama-Birmingham, Birmingham, AL 35294, USA; E-Mail: ghunter@uab.edu; 2 Department of Physical Therapy, University of Alabama-Birmingham, Birmingham, AL 35294, USA; E-Mail: bickel@uab.edu

**Keywords:** combined exercise training, inflammation, hypertrophy, elderly

## Abstract

The purpose of this investigation was to determine if differences in inflammatory cytokines exist between fat-free mass responders *versus* non-responders following a combined resistance/aerobic training program in older women. Fifty women over 60 years old, mean BMI 27 ± 4 (kg/m^2^) and physically untrained, participated in a combined training program for 16-weeks. Body composition, muscle strength, and serum inflammatory markers (TNF-α, CRP, and IL-6) were assessed at baseline and 16-weeks post-training. A significant time effect was observed for weight, %fat, fat mass, and all strength measures (*p* < 0.05). A group interaction was observed for TNF-α (*p* < 0.05), which revealed higher concentrations of circulating TNF-α at baseline (18%) and post-exercise training (24%) in non-responders compared to responders (*p* < 0.05). In conclusion, this study revealed a significantly greater concentration of circulating TNF-α in older women that do not increase fat-free mass following training.

## 1. Introduction

Aging is associated with a decline in skeletal muscle mass, muscle strength, and functional capacity [1]. It has been well documented that progressive resistance training is able to blunt the age-related loss of skeletal muscle mass, and increase muscle strength and cross-sectional area in older adults [1,2,3]. Additionally, while several early studies revealed that combining strength training with endurance training can compromise strength development [4,5,6], more recent investigations in older adults have shown that combined training can lead to similar or greater gains in muscular strength compared to resistance training alone [7,8,9]. Furthermore, we recently demonstrated that a combined training program of three different doses led to similar improvements in strength and functional tasks in older women [10].

Skeletal muscle adaptations to strength training can be influenced by factors such as age, nutrition, genetics, and other factors that can influence muscle protein turnover (muscle protein synthesis and breakdown); therefore, variable responses to exercise training are often observed within and between studies [11,12,13]. While resistance training alone or combined aerobic and resistance training have consistently yielded improvements in muscular strength and endurance in older adults [1,8,10,14], there are often large individual differences in the skeletal muscle hypertrophic responses to training [11,13,15]. Therefore, it is important to identify potential mechanisms responsible for these individual differences in muscle mass gains.

Elevated levels of inflammatory cytokines (TNF-α, IL-6, and CRP) are thought to play a critical role in the functional decline of older adults. Cross-sectional studies have demonstrated that higher levels of inflammatory cytokines are associated with lower muscle mass and strength [16,17]. Furthermore, clinical and animal experimental studies have shown a relationship between elevated inflammation and loss of muscle mass, strength, and greater muscle breakdown [17,18]. Additionally, baseline levels of sTNFR-I were inversely associated with muscle strength in frail older adults following a 12-week resistance training program [19]. Therefore, it seems plausible that baseline inflammatory levels may blunt training induced gains in muscle mass by disrupting signaling cascades associated with protein synthesis and/or degradation during training. Our objective was to determine if differences in inflammatory cytokines exist between fat-free mass responders *versus* non-responders following a combined resistance/aerobic training program in older women.

## 2. Experimental Section

### 2.1. Subjects

Subjects were 63 postmenopausal women over 60 years of age (Table 1). Subjects had a BMI < 30 kg/m^2^ and were physically untrained (self-reporting no exercise training at initial screening). Women with a BMI > 30 kg/m^2^) or engaging in > 2 h of structured exercise or already performing weight training exercise or routine walking were excluded. Preliminary screening for study inclusion included a physical examination, dual-energy X-ray absorptiometry (DEXA), and a 12 lead EKG. Participants were excluded from the study if they were hypertensive, displayed any abnormal EKG responses at rest or during exercise, or DEXA assessment revealed osteoporosis. Subjects had no history of heart disease or diabetes mellitus, were non-smokers, and not taking medications known to affect energy expenditure, insulin level, heart rate, or thyroid function. The study was approved by the Institutional Review Board. All women provided informed consent prior to participation.

**Table 1:** Descriptive statistics and outcome variables pre- and 16 weeks post-training by responders *vs.* non-responders.

	Responders (*n* = 30)		Non-Responders (*n* = 20)		G	T	G*T
	Pre	Post	Pre	Post	*p*	*p*	*p*
Age (years)	64.8 ± 3.6	65.2 ± 3.6	64.6 ± 3.2	65.1 ± 3.2	0.879	0.001	0.368
Weight (kg)	72.1 ± 9.7	71.8 ± 9.7	75.1 ± 14.1	73.5 ± 11.6	0.468	0.025	0.112
BMI (kg/m^2^)	26.8 ± 3.9	26.6 ± 4.1	27.9 ± 3.3	27.4 ± 3.7	0.398	0.011	0.154
%fat	43.1 ± 5.7	40.9 ± 5.9	41.5 ± 7	42.4 ± 5.9	0.974	0.013	0.001
Fat Mass (kg)	31.1 ± 7.9	29.7 ± 8.0	32.4 ± 12.2	32.0 ± 9.5	0.493	0.045	0.240
Fat-Free Mass (kg)	40.9 ± 3.6	42.2 ± 3.6	42.7 ± 3.5	41.5 ± 3.2	0.610	0.827	0.001
TNF-α (pg/mL)	6.1 ± 2.3	5.9 ± 2.1	7.3 ± 1.9	7.5 ± 2.3	0.022	0.718	0.239
IL-6 (pg/mL)	1.9 ± 0.91	1.6 ± 0.70	2.0 ± 2.3	2.2 ± 2.8	0.477	0.758	0.074
CRP (mg/L)	3.2 ± 3.7	2.8 ± 2.9	2.6 ± 2.1	2.8 ± 2.7	0.715	0.772	0.344
Chest Press (kg)	23.6 ± 6.4	27.1 ± 8.1	22 ± 3.9	27.4 ± 6.2	0.688	0.001	0.226
Shoulder Press (kg)	23.8 ± 4.9	26.2 ± 5.4	23.4 ± 3.9	26.9 ± 4.4	0.868	0.001	0.177
Leg Press (kg)	87.9 ± 26.1	110.7 ± 34.5	90.7 ± 16.3	111.8 ± 21.2	0.790	0.001	0.726
Knee Extension (kg)	30.6 ± 8.9	38.1 ± 10	30.9 ± 6.3	35.8 ± 6.4	0.670	0.001	0.114
VO_2max_ (mL/kg/min)	23.4 ± 4.6	23.9 ± 4.9	21.8 ± 4.8	22.7 ± 4.6	0.379	0.172	0.665

G = intervention group, T = time; G*T = group × time interaction. All data are presented as means ± standard deviations. Statistically different within group means are represented by different superscripts.

### 2.2. Study Design

Subjects were randomly assigned to one of three exercise training groups. Group 1 performed one resistance exercise training (RET) and one aerobic exercise training (AET) session per week; Group 2 performed two RET and two AET sessions per week; and Group 3 performed three RET and three AET sessions per week. Group 1 trained two times each week (one aerobic and the other resistance) on non-consecutive days, Group 2 trained 3 to 4 days each week (two aerobic and two resistance) and Group 3 trained between 4 and 6 days each week (3 aerobic and 3 resistance). In order to improve adherence, subjects in Group 2 and Group 3 were allowed to have combined aerobic and resistance training sessions on the same day. Subjects in Group 2 were allowed one combined training session each week and subjects in Group 3 were allowed two combined training sessions each week. Both Group 2 and 3 were required to have at least one training session each week in which only aerobic and only resistance training are performed. Muscular strength, cardiovascular fitness, and body composition measurements were made pre- and 16-weeks post-training. No significant differences were observed between the three training groups (see previously published manuscript) [10]; therefore, data were pooled in this study to assess responders (any gain in fat-free mass) and non-responders (no increase in fat-free mass).

### 2.3. Strength Testing

Two exercise sessions within the first week were used to familiarize the subjects with the movement patterns of the exercise prior to pre-training strength measurement. The maximum amount of weight that could be lifted once (1 RM) was measured for leg press, knee extension, chest press, and shoulder press using previously reported methods [20]. Briefly, after a warm-up subjects lifted progressively heavier weights until 2 consecutive failures occurred. Coefficient of variability for 1 RM testing in our lab is less than 3%.

### 2.4. Aerobic Capacity Testing

Maximal aerobic exercise testing was physician-supervised and conducted using the modified Balke treadmill test protocol. A metabolic cart, calibrated prior to testing (Max-1 Cart, Physio-Dyne Instrument Corporation, Quogue, NY, USA), was used to evaluate ventilatory expired gases. Monitoring consisted of 12-lead electrocardiogram and blood pressure measurements taken every two min (Omron Blood Pressure Monitor, model HEM-780; Omron Healthcare, Inc., 1200 Lakeside Dr. Bannockburn, IL, USA).

### 2.5. Resistance and Aerobic Training Program

All exercise sessions were supervised by an exercise physiologist. The duration of the resistance exercise sessions was approximately 60 min, including a brief warm up. Each exercise session began with a 3 min warm-up on either a treadmill or cycle ergometer at low intensity. Subjects performed the following resistance training exercises: squats, leg press, leg extension, hamstring curl, bench press, military press, elbow flexion, and triceps extension. Subjects began the resistance training program with 1 set of 10 repetitions at an intensity of 60% of their one repetition maximum (1 RM) and progressed to 2 sets of 10 repetitions at 80% of 1 RM by week 6.

Subjects performed aerobic exercise on a treadmill or cycle ergometer (95% of aerobic exercise was performed on a treadmill). Subjects trained initially for 20 min at 65% of maximum heart rate (MHR), and progressively increased their training until they were able to exercise continuously for 40 min at 80% of MHR. Heart rate (HR) was monitored throughout each session by a Polar Vantage XL HR monitor (Polar Beat, Port Washington, NY, USA).

### 2.6. Laboratory Analyses

Blood samples were obtained at baseline following an overnight fast and 24 h following completion of the last exercise session. Inflammatory markers were assessed using enzyme-linked immunosorbent assays (ELISAs). All samples were analyzed in duplicate. TNF-α was analyzed using the high-sensitivity ELISA kit (Quantikine HSTA00C, R&D Systems, Minneapolis, MN, USA). IL-6 was assayed using the high sensitivity ELISA kit (Quantikine HS600B, R&D Systems, Minneapolis, MN, USA). CRP was assayed with the high-sensitivity ELISA kit (030-9710s, ALPCO, Windham, NY, USA).

### 2.7. Body Composition Measurements

Body composition (total fat mass and total fat-free mass) was measured by DEXA (Prodigy; Lunar Radiation, Madison, WI, USA). The whole body scans were analyzed with the use of ADULT software, version 1.33 (Lunar Radiation).

### 2.8. Statistical Analysis

Descriptive statistics were computed at baseline and 16-weeks post-training (means ± standard deviations) All statistical models were evaluated for residual normality and log transformed when appropriate. There were no significant group or group × time interactions for the three training groups; therefore data were pooled for analyses (previously published data) [10]. Individuals were divided into two groups: responders (any gain in fat-free mass) and non-responders (no increase in fat-free mass). Pearson’s correlation analyses were performed to identify associations between outcome variables. Overall comparisons of the changes in body composition, strength, and circulating inflammatory markers were performed using a one-way ANOVA with repeated measures. All data were analyzed using the Statistical Package for the Social Sciences (SPSS, version 19.0, Chicago, IL, USA).

## 3. Results

**Figure 1:**
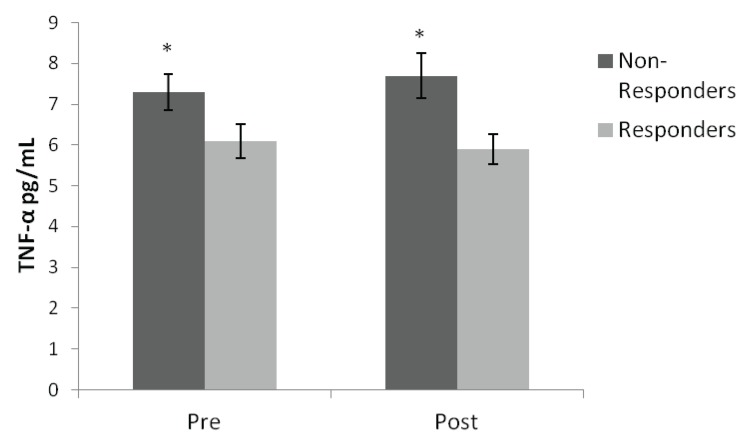
* Significantly greater concentration of circulating TNF-α in fat-free mass non-responders compared to responders (*p* < 0.05).

Descriptive statistics at baseline and the effects of time, group, and group × time interactions for each variable are shown in Table 1. A significant time effect was observed for weight, %fat, fat mass, and all strength measures (*p* < 0.05), demonstrating an improvement following training. As expected a group × time interaction was found for %fat and fat-free mass, such that responders decreased %fat and increased fat-free mass while non-responders increased %fat and decreased fat-free mass (*p* = 0.001). A group interaction was observed for TNF-α (*p* < 0.05). Specifically, there was a significantly higher concentration of circulating TNF-α at baseline (18%) and post-exercise training (24%) in non-responders compared to responders (*p* < 0.05) (Figure 1). While there were distinct differences between circulating TNF-α concentration between non-responders and responders, no significant correlations were found between cytokines and other dependent variables. Additionally, no significant changes were observed for IL-6, CRP, and aerobic capacity. There was a non-significant trend (*p* = 0.074) for a group × time interaction for IL-6, such that non-responders demonstrated an increase whereas responders demonstrated a decrease in IL-6.

## 4. Discussion and Conclusions

All women had significant improvements in skeletal muscle strength, however, 40% of the older women in this study did not have any positive gains in fat-free mass following 16-weeks of combined training. We believe this is the first report to reveal a significantly greater concentration of circulating TNF-α in older women that do not increase fat-free mass following 16-weeks of combined training. While we do not have skeletal muscle biopsies to elucidate potential mechanisms within the muscle, this observation is important and suggests that systemic inflammation may be an important factor that interferes with skeletal muscle hypertrophy during exercise training.

While the exact substances responsible for interference in skeletal muscle remodeling remains to be determined, low-grade chronic systemic inflammation is thought to be a major contributor [17,21]. To our knowledge, this is the first longitudinal study to demonstrate higher circulating TNF-α in the untrained and trained state of older women that had no increases in fat-free mass as compared to older women that increased fat-free mass following combined training. There have been a number of longitundal studies demonstrating the powerful effects of weight loss, from both diet and exercise, for reducing systemic inflammation [22,23]. However what is less known is the role of changes in skeletal muscle mass on systemic inflammation. A recent investigation by Mavros *et al.* (2014) found that reductions in CRP in older adults was associated with increases in skeletal muscle mass following a 12-month resistance training program, which suggests that skeletal muscle mass may also be playing a critical role in regulation of systemic inflammation [24]. While this is the first longitudinal study published to date demonstrating this relationship, cross sectional studies have shown inverse associations between IL-6 and TNF-α with limb mass and strength [17]. Furthermore, while we did not find any independent associations between any of our inflammatory markers and skeletal muscle mass or strength, the fact that our non-responders had higher concentrations of TNF-α at all time points measured suggests the possibility that chronically elevated circulating concentrations of TNF-α may disrupt the regeneration of muscle tissue in older women. The effects of TNF-α have been shown to be concentration and duration dependent, such that low concentrations act as a mediator of growth and regeneration while high concentrations and prolonged exposure can impair muscle regeneration [25,26]. Furthermore, it is now widely accepted that exercise can induce both pro- and anti-inflammatory responses in skeletal muscle, which can be further influenced by the mode, intensity, and duration of exercise [27]. While we can only speculate as to why the observed relationship between TNF-α and muscle mass existed, it is possible that women in this study with elevated basal concentrations of TNF-α had an exacerbated TNF-α response following exercise training that exceeded the threshold of cytokine regulation of muscle synthesis in favor of degradation.

In conclusion, this study revealed a significantly greater concentration of circulating TNF-α in older women that do not increase fat-free mass following 16-weeks of combined training. This suggests that systemic inflammation may be an important factor that disrupts skeletal muscle hypertrophy during exercise training. Future longitudinal studies conducted in humans are needed to establish cause and effect and identify precise mechanisms in which systemic inflammation can alter skeletal muscle remodeling.
